# Gastric Kaposi Sarcoma With Distinct “Lobster‐Like” Endoscopic Lesions in a Human Immunodeficiency Virus‐Positive Patient

**DOI:** 10.1002/deo2.70256

**Published:** 2025-11-25

**Authors:** Seyed Ali Safizadeh Shabestari, Aidin Farahvash, Mohammad Jafar Farahvash

**Affiliations:** ^1^ Southend University Hospital Mid and South Essex NHS Foundation Trust Westcliff‐on‐Sea England; ^2^ Psychiatry Department Tehran University of Medical Sciences Tehran Iran; ^3^ Tehran University of Medical Sciences Tehran Iran

**Keywords:** Gastrointestinal diseases, HIV infections, Human Herpesvirus‐8, Kaposi Sarcoma, Stomach neoplasms

## Abstract

Kaposi Sarcoma (KS) is a rare vascular tumor linked to Human Herpesvirus‐8 (HHV‐8) infection, most often affecting immunocompromised patients with Human Immunodeficiency Virus/Acquired Immunodeficiency Syndrome (HIV/AIDS). Gastrointestinal KS (GI‐KS) is frequently underdiagnosed, particularly in resource‐limited settings, and may present with nonspecific symptoms. We describe a 52‐year‐old HIV‐positive male with advanced immunosuppression (CD4 count: 34 cells/µL, viral load: 236,670 copies/mL) who presented with abdominal pain, weight loss, and violaceous cutaneous lesions. Upper GI endoscopy revealed multiple reddish nodular gastric lesions with a distinctive “lobster‐like” morphology. Histopathology showed abnormal vascular proliferation, with endothelial cells positive for CD34 and HHV‐8, confirming gastric KS. Colonoscopy was unremarkable. The patient received HAART only, without systemic chemotherapy, and refused further treatment and follow‐up. The coexistence of gastric and cutaneous KS in this patient reflects disseminated disease and highlights the importance of early endoscopic evaluation in HIV‐infected individuals with unexplained GI complaints. This case adds to the limited literature on gastric KS in the Middle East and documents an unusual endoscopic appearance that may aid in earlier recognition. To our knowledge, this is the first report of a gastric KS lesion with a novel “lobster‐like” appearance. This appearance likely reflects the tumor's vascular origin, producing bilateral, claw‐shaped mucosal elevations due to submucosal vascular proliferation. Greater awareness of such presentations can facilitate timely diagnosis, multidisciplinary management, and improved outcomes in advanced HIV.

## 1 Introduction

Kaposi Sarcoma (KS) is a rare, multifocal vascular tumor associated with Human Herpesvirus‐8 (HHV‐8) infection. It most commonly occurs in immunocompromised individuals, particularly in those with Human Immunodeficiency Virus/Acquired Immunodeficiency Syndrome (HIV/AIDS). There are four epidemiological forms of KS: classic, endemic (African), iatrogenic, and epidemic (AIDS‐related) [1, 2]. Among them, the epidemic KS is the most prevalent in HIV‐infected patients and can affect various organs, including the skin, lymph nodes, lungs, and the gastrointestinal (GI) tract [1, 2].

The GI manifestations of KS are of particular concern because they can lead to severe complications such as hemorrhage, obstruction, and perforation if left untreated [2, 3]. This case report highlights a patient in Iran with HIV infection and gastric KS, emphasizing the diagnostic challenges associated with this condition.

### 1.1 Case Presentation

The patient is a 52‐year‐old Iranian male who presented to the GI clinic with complaints of stomach pain, general malaise, and significant weight loss of 10 kg over the past month.

On physical examination, his vital signs were within normal limits. He appeared cachectic and exhibited marked tenderness in the epigastric region. Although the patient was in obvious distress due to abdominal pain, there were no signs of peritoneal irritation, such as rebound tenderness or guarding.

**FIGURE 1:**
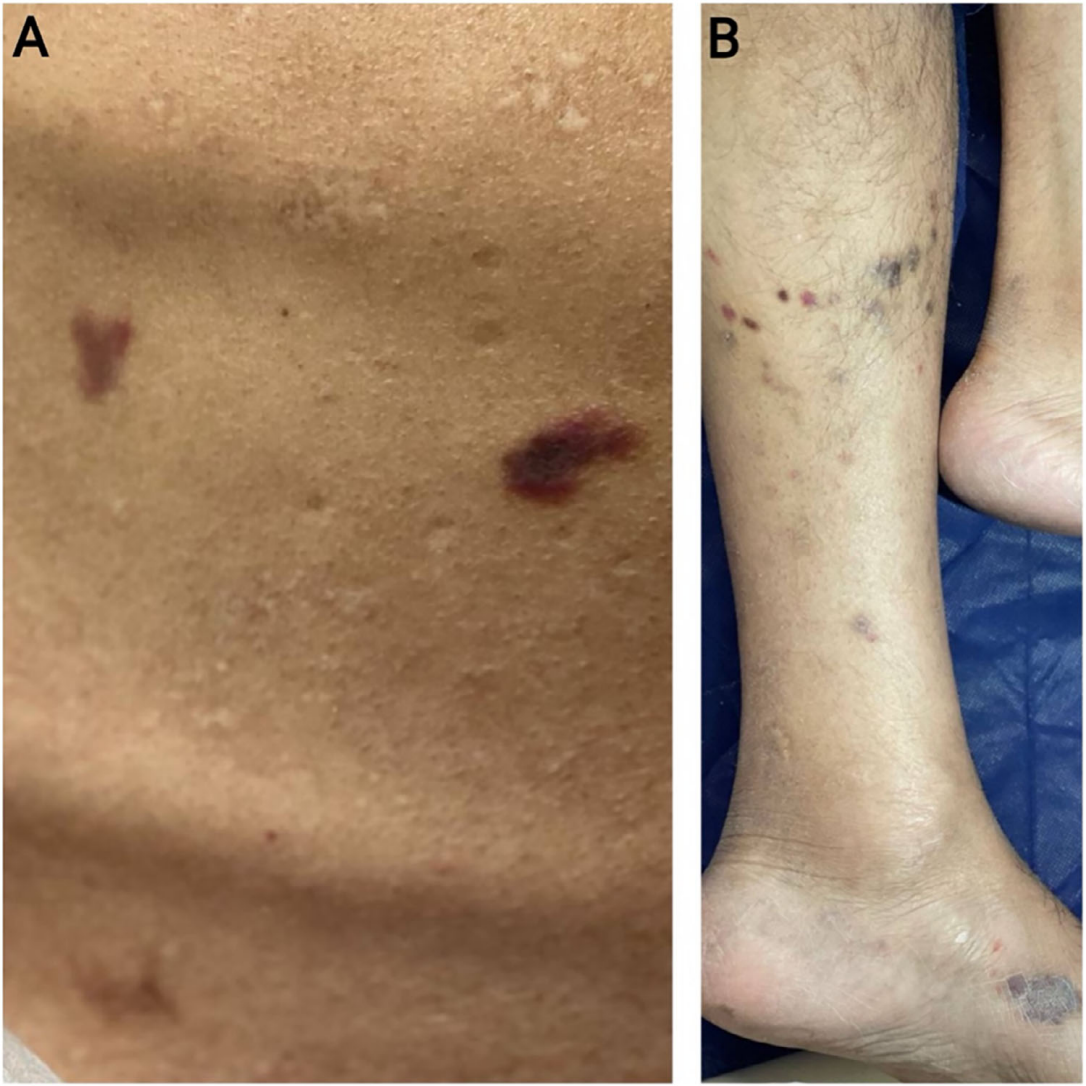
Cutaneous Kaposi Sarcoma. Violaceous to dark red nodules and patches on the patient's back (A) and lower extremities (B), with prominent lesions around the ankles and lower legs, typical of the disease in immunocompromised patients.

On skin examination, there were violaceous to dark red nodules and patches on the patient's back (Figure 1A) and lower extremities (Figure 1B), with prominent lesions around the ankles and lower legs.

The patient received HAART as sole therapy, without systemic chemotherapy. Follow‐up endoscopy was not undertaken, and initial investigations revealed no other GI lesions.

### 1.2 Investigations

**FIGURE 2:**
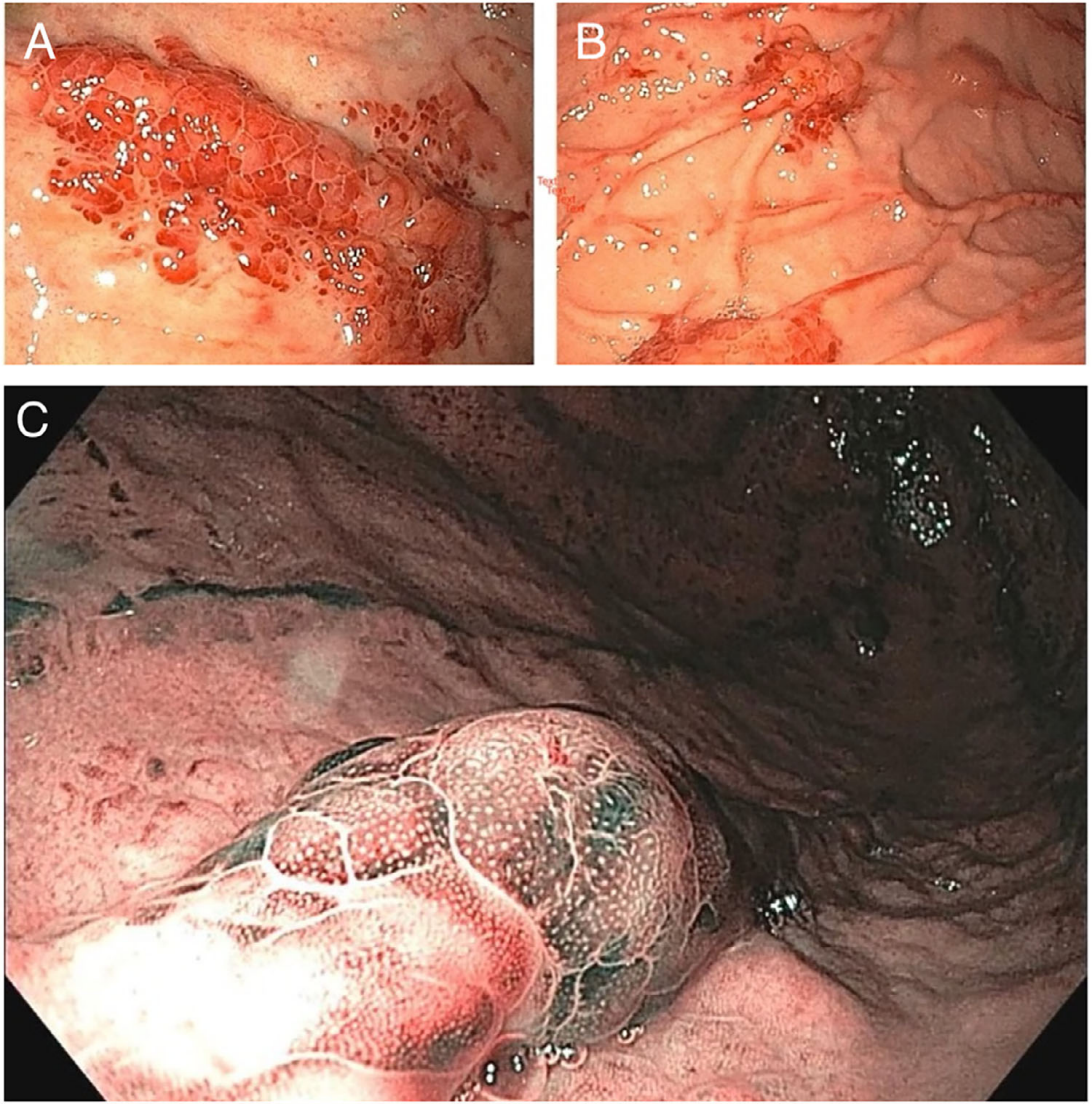
Gastric Kaposi Sarcoma: Lobster‐like Lesions. (A) Upper gastrointestinal endoscopy demonstrating a raised, reddish plaque‐like lesion with a distinctive “lobster‐like” configuration, located along the greater curvature of the gastric antrum. (B) Endoscopic view showing multiple raised nodular lesions on the gastric mucosa, similarly situated on the greater curvature of the antrum, consistent with gastric Kaposi sarcoma. (C) Narrow‐band imaging (NBI) endoscopy showing a nodular gastric lesion with prominent vascular markings, characteristic of Kaposi sarcoma. The enhanced imaging clearly delineates the disorganized vasculature, aiding diagnostic recognition.

**FIGURE 3:**
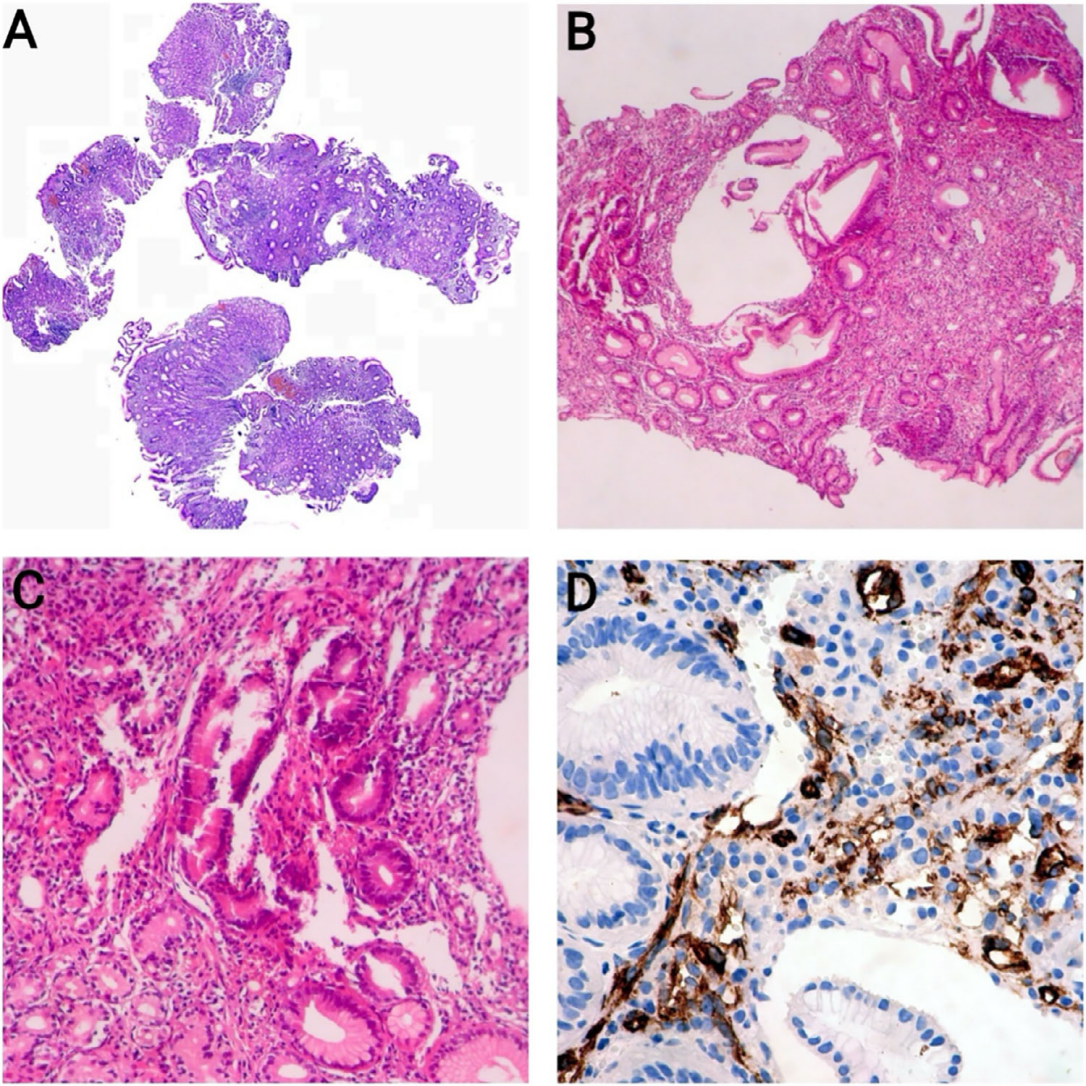
Histopathological and Immunohistochemical Findings. (A) Low‐power H&E section showing gastric mucosa with mild chronic gastritis, slight lymphoplasmacytic infiltration, and RBC extravasation (magnification ×40). (B, C) Higher magnification H&E sections demonstrating regenerative changes in foveolar cells, with no significant vascular proliferation discernible in the lamina propria (magnifications ×100 and ×200). (D) Immunohistochemical staining for CD34 (magnification ×400) reveals proliferation of capillary‐sized vascular channels and small aggregates of endothelial cells.

An upper GI endoscopy was performed due to his complaints of abdominal discomfort. It revealed multiple reddish nodular lesions in the stomach (Figure 2). These unusual lesions were characterized by distinct morphology, resembling lobster claws, leading to their designation as “Lobster‐like Erosions.” Biopsies were taken from these lesions for histopathological analysis (Figure 3). A colonoscopy was performed, and no other GI lesions were identified.

Laboratory investigations revealed normal liver function tests and normal serum amylase levels. Screening for autoimmune markers, including rheumatoid factor, anti‐CCP, ANA, and anti‐double‐stranded DNA antibodies, and real‐time polymerase chain reaction (RT‐PCR) tests for coronavirus disease 2019 and Influenza were all negative. However, a CD4 count of 34 cells/µL, a CD4/CD8 ratio of 0.026, and an HIV PCR (viral load) of 236,670 copies/mL were found, indicating advanced HIV infection.

The microscopic examination of the gastric biopsy revealed fragments of oxyntic and non‐oxyntic gastric mucosa with intact foveolar cells (Figure 3A). The lamina propria was notably infiltrated by polymorphic lymphoid cells (Figure 3B,C). Abnormal vascular architecture was observed, with small capillary‐sized vessels demonstrating positive staining for CD34 (Figure 3D), a marker for endothelial cells. Immunohistochemical (IHC) staining further confirmed the presence of Human Herpesvirus 8 (HHV8), a key marker for the pathologies observed in the tissue sample. Moreover, Giemsa staining showed the absence of *Helicobacter pylori*, ruling out *H. pylori*‐associated gastritis.

The IHC findings, that is, the co‐expression of CD34 and HHV8 in endothelial cells, the proliferation of capillary‐size vessels, combined with the presence of HHV8, all supported the diagnosis of gastric KS.

## 2 Discussion: GI Involvement in KS

GI‐KS is a significant concern in patients with HIV/AIDS, especially in the era of effective antiretroviral therapy, as presented in our case. Older studies reported GI involvement in up to half of untreated HIV/AIDS patients, but recent data indicate GI‐KS is rare; a 10‐year endoscopy series found GI‐KS in only ∼1.9% of HIV‐positive patients undergoing upper endoscopy [4]. Among those with GI‐KS, the stomach is involved in approximately 55% of cases [4], making it one of the most frequently affected sites in the GI tract. Clinically, GI involvement may manifest with a wide spectrum of presentations, ranging from mild nonspecific symptoms such as abdominal discomfort, early satiety, or diarrhea, to severe and life‐threatening complications including GI hemorrhage, bowel obstruction, or perforation [1, 2, 4, 5]. Many patients remain asymptomatic, making early detection challenging. In this case, the patient's weight loss, asthenia, and abdominal discomfort prompted further investigation, revealing GI involvement.

GI‐KS can occur at any location from the oral cavity to the rectum, though it is most commonly found in the stomach, small intestine, and colon [1, 2], with esophageal lesions being less frequent [5, 6].

Endoscopic findings range from flat macules to nodules, polypoid masses, and ulcerated “volcano‐like” or bull's‐eye lesions. [2, 3, 4, 5, 10, 11] Infiltrative lesions may mimic submucosal tumors. Histology typically shows spindle‐cell proliferation forming vascular spaces [1, 5]. Because KS primarily involves the submucosa, superficial biopsies may be falsely negative, making adequate tissue sampling essential [10]. Immunohistochemistry is indispensable, with HHV‐8 staining showing near‐perfect sensitivity and specificity, distinguishing KS from mimics such as GI stromal tumor (GIST) or angiosarcoma. Our case highlights a previously undescribed “lobster‐like” morphology, expanding the spectrum of GI‐KS appearances. The “lobster‐like” appearance likely reflects the tumor's vascular origin, producing bilateral, claw‐shaped mucosal elevations due to submucosal vascular proliferation. This underscores the importance of biopsying suspicious lesions in immunocompromised patients, even when the morphology is atypical. Table 1 summarizes the spectrum of endoscopic features reported in gastric KS. The diagnosis of GI‐KS relies heavily on immunohistochemistry, with HHV‐8 staining serving as a reliable and specific marker [10]. This is critical for distinguishing KS from other spindle‐cell lesions. Management focuses on HAART, which often leads to regression of KS lesions by restoring immune function [1, 2]. In patients with disseminated or symptomatic visceral disease, systemic chemotherapy, typically liposomal doxorubicin, may be required [12]. In this case, the patient declined chemotherapy and was managed with HAART alone, highlighting the practical challenges when patients are unable or unwilling to pursue comprehensive therapy. This case emphasizes the importance of early recognition, comprehensive GI evaluation, and multidisciplinary care involving gastroenterology, infectious disease, and oncology. Recognition of novel morphologies such as “lobster‐like” erosions may support timely diagnosis and improve outcomes in advanced HIV disease.

**TABLE 1:** Reported endoscopic features of gastric Kaposi sarcoma in the literature.

Morphology	Description/Examples	References
Flat/maculopapular lesions	Small red macules or patches	Weprin et al. (1982) [9]
Polypoid/nodular lesions	Raised, darker nodules may ulcerate	Carmo et al. (2017) [8]; Rezende et al. (2016) [4]
Volcano‐like/bull's‐eye lesions	Central umbilication, ulcerated center	Lee et al. (2015) [11]; Nagata et al. (2012) [10]
Infiltrative or SMT‐like lesions	Submucosal tumor‐like appearance	Taccogna et al. (2009) [12]
Hemorrhagic/ulcerated lesions	Friable, prone to bleeding	Multiple sources [12, 13]
“Lobster‐like” erosions (our case)	Distinct bilateral claw‐like appearance	Present case

## Author Contributions

**Seyed Ali Safizadeh Shabestari** contributed to conceptualization, methodology, data curation, and investigation. He was responsible for visualization and drafting the original manuscript. He also participated in reviewing and editing the manuscript. **Aidin Farahvash** contributed to reviewing and editing the manuscript. **Mohammad Jafar Farahvash** contributed to conceptualization, methodology, data curation, and investigation. He was responsible for validation and supervision, and he contributed to reviewing and editing the manuscript.

## Funding

The authors have nothing to report.

## Conflicts of Interest

The authors declare no conflicts of interest.
